# Molecularly imprinted nanogels as synthetic recognition materials for the ultrasensitive detection of periodontal disease biomarkers

**DOI:** 10.1007/s00216-024-05395-6

**Published:** 2024-06-20

**Authors:** Thomas Hix-Janssens, Julia R. Davies, Nicholas W. Turner, Börje Sellergren, Mark V. Sullivan

**Affiliations:** 1 Department of Biomedical Science, Faculty of Health and Society, Malmö University, 205 06 Malmö, Sweden; 2 Section for Oral Biology and Pathology, Faculty of Odontology, Malmö University, 205 06 Malmö, Sweden; 3 Department of Chemistry, Dainton Building, University of Sheffield, Brook Hill, Sheffield, S3 7HF UK

**Keywords:** Molecularly imprinted polymers, Nanogels, Periodontal disease, Surface plasmon resonance

## Abstract

Periodontal disease affects supporting dental structures and ranks among one of the top most expensive conditions to treat in the world. Moreover, in recent years, the disease has also been linked to cardiovascular and Alzheimer’s diseases. At present, there is a serious lack of accurate diagnostic tools to identify people at severe risk of periodontal disease progression. *Porphyromonas gingivalis* is often considered one of the most contributing factors towards disease progression. It produces the Arg- and Lys-specific proteases Rgp and Kgp, respectively. Within this work, a short epitope sequence of these proteases is immobilised onto a magnetic nanoparticle platform. These are then used as a template to produce high-affinity, selective molecularly imprinted nanogels, using the common monomers *N*-tert-butylacrylamide (TBAM), *N*-isopropyl acrylamide (NIPAM), and N-(3-aminopropyl) methacrylamide hydrochloride (APMA). *N*,*N*-Methylene bis(acrylamide) (BIS) was used as a crosslinking monomer to form the interconnected polymeric network. The produced nanogels were immobilised onto a planar gold surface and characterised using the optical technique of surface plasmon resonance. They showed high selectivity and affinity towards their template, with affinity constants of 79.4 and 89.7 nM for the Rgp and Kgp epitope nanogels, respectively. From their calibration curves, the theoretical limit of detection was determined to be 1.27 nM for the Rgp nanogels and 2.00 nM for the Kgp nanogels. Furthermore, they also showed excellent selectivity against bacterial culture supernatants E8 (Rgp knockout), K1A (Kgp knockout), and W50-d (wild-type) strains in complex medium of brain heart infusion (BHI).

**Supplementary Information:**

The online version contains supplementary material available at 10.1007/s00216-024-05395-6.

## Introduction

Periodontal disease (PD) is an oral health condition that affects the supporting dental structures, such as alveolar bone and connective tissues [1]. On top of that, they rank among the top most expensive conditions to treat in the world and around 7% of the adult population suffers from severe disease with a high risk of tooth loss [2]. Moreover, in recent years, the disease has also been linked to cardiovascular and Alzheimer’s diseases [3, 4]. The disease is characterised by buildup of dental plaque and inflammation in the surrounding tissues [5]. Early-stage periodontal disease, gingivitis, is characterised by red and swollen gums. In some individuals, bacteria in the gingival biofilm secrete an excess of proteases that affect surrounding tissues [6]. An immune response is then likely to trigger the recruitment of inflammatory response units such as cytokines and matrix metalloproteases. These, in turn, lead to further destruction of soft tissues and alveolar bone [7].

At present, there is a serious lack of accurate diagnostic tools to identify persons at risk of progression from gingivitis to severe periodontal disease with risk for eventual tooth loss. This results both in a tendency for overtreatment of some patients, while others may be missed [8]. Currently, there are four main methods to diagnose periodontal disease: inspection, palpation, probing, and taking radiographic images [8]. However, these methods are often subjective and can lead to misdiagnosis or diagnostic errors. Biosensors hold the potential as viable platforms for accurate diagnosis of disease progression so that patients are individualised and given appropriate treatment. The principle behind biosensors is that a biological recognition event is turned into a measurable signal output [9]. Many biological events can be used, such as enzymatic degradation or binding between ligand and receptor. A transducer is used to change the biorecognition event into a signal output. These can be based on optical, electrochemical, or acoustic methods that are based on a change in, for example, mass, current, or refractive index [10].

Traditionally, natural recognition materials such as antibodies or enzymes are used for the specific detection of target analytes in biosensors because of their high degree of target selectivity and specificity [11]. However, these natural recognition materials often suffer from high manufacturing costs, short shelf-life, and poor stability, resulting in degradation through changes in environmental conditions (temperature and pH) with subsequent denaturation of the recognition material leading to function impairment [12, 13]. This has led to the use of cost-effective synthetic recognition material alternatives as they can offer increased stability and robustness that is lacking from their biological counterparts.

Molecularly imprinted polymers (MIPs) are a synthetic option that have displayed capability as a viable alternative to antibodies and enzymes, because of the ease of production, low cost, high affinity, and robustness in the extremes of pH and temperature [14]. Using a self-assembly approach, MIPs are generally produced firstly with functional monomers, pre-organised around a template molecule (target analyte) with non-covalent interactions, such as hydrogen bonding, van der Waals, ionic interactions, and hydrophobic bonding forming the monomer-template complex. Next, in the presence of a suitable crosslinker and after initiating the polymerisation reaction, the monomers polymerise around the template, encapsulating the latter within a polymeric matrix. After subsequent removal of the template, molecular recognition cavities are left within the polymer, which are specific to the shape and size of the template used and, in addition, complement substructures of macromolecular targets [15, 16]. Following a quasi-generic protocol, polyacrylamide gel-based nanoMIPs (nanogels) can now be manufactured to target a wide range of biomolecules. Similar to antibodies, these binders can be affinity purified, leading to an overall enhanced, nearly monoclonal affinity, qualifying these receptors for a wide range of biomedical applications [17, 18]. This progress stems from recent advances in polymer, colloid, and host–guest chemistry, particularly through the application of epitope imprinting [19] combined with solid-phase [20] or magnetic dispersed phase [17, 21]. Furthermore, using this synthesis methodology permits engineering of receptors exhibiting one binding site per nanogel particle, allowing for the ease and use of mathematical modelling functions that are commonly employed with biological recognition counterparts [22]. Moreover, nanoMIPs are well suited for their use as recognition elements in chemical sensors [23, 24]. Their sturdiness combined with a simple and reproducible synthesis procedure gives these synthetic receptors an edge over labile biologically derived receptors.

**Fig. 1:**
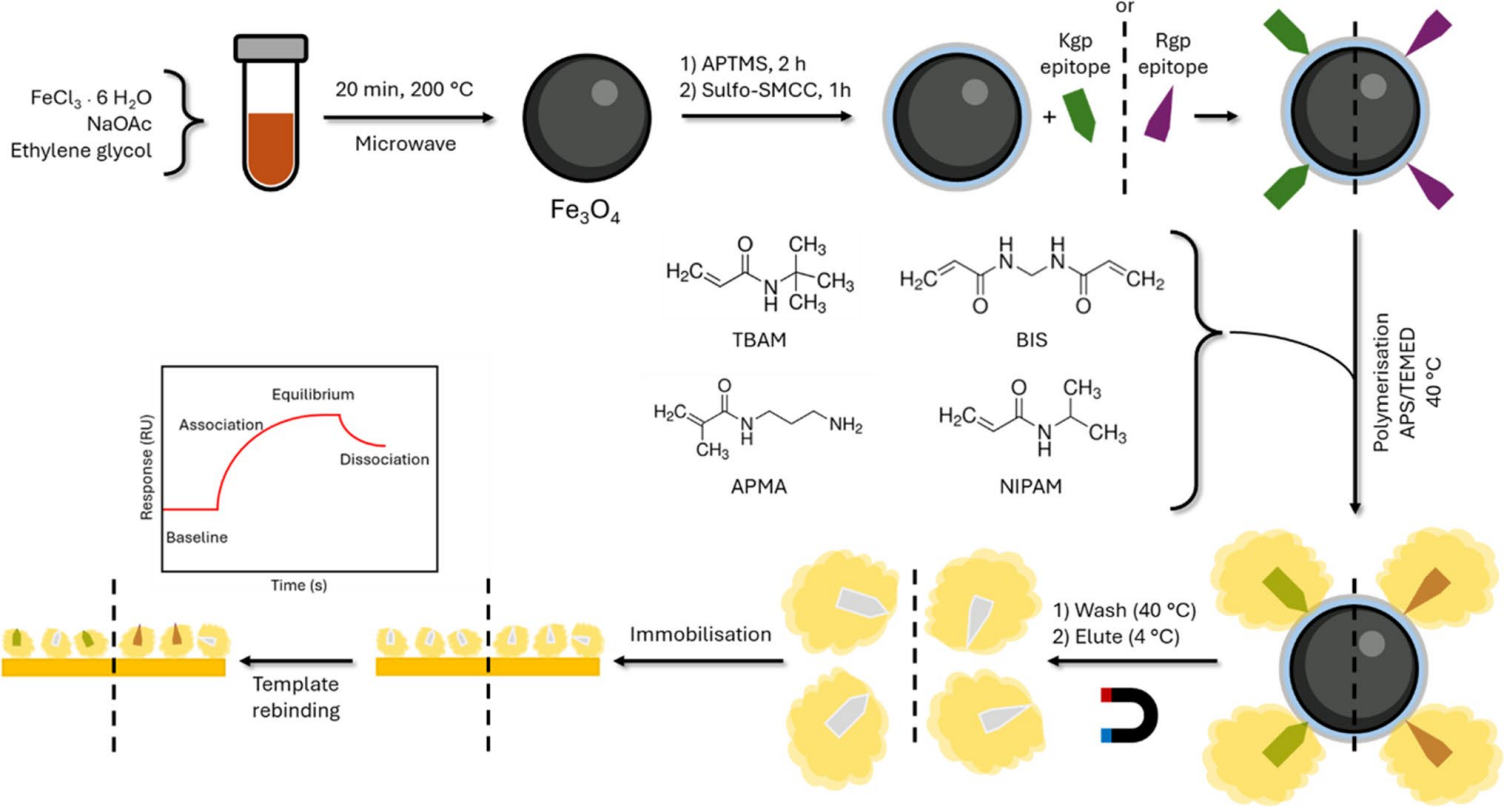
Magnetic (Fe_3_O_4_) nanoparticles are prepared using a microwave. After modification using APTMS, the Kgp or Rgp epitopes are immobilised onto the surface through linking with sulfo-SMCC. MIP nanogels are polymerised around the template using the APS and TEMED couple, at 40 °C. After elution of nanogels, they are immobilised onto a dextran-modified SPR chip using EDC/NHS coupling. Rebinding of target templates results in a signal change. The potential number of imprinting points on the magnetic (Fe_3_O_4_) nanoparticle is unknown and the image is only meant to highlight that there are potentially multiple imprinting points and not a definitive number

One of the bacteria present in the oral microbiome is *Porphyromonas gingivalis* (*PG*), an anaerobic Gram-negative pathogen implicated in PD [25]. They produce the Gingipains Rgp and Kgp, which are ca 50-kDa cysteine proteases with different cleavage preferences, Rgp favouring hydrolysis at Arg-Xaa sites, while Kgp prefers Lys-Xaa sites. In our previous work, we have explored microcontact imprinting based on recombinantly produced protein templates to quantify the expression level of proteases produced by *P. gingivalis* [26]. Given the lack of access to such templates, we have here exploited epitope imprinting. Epitopes corresponding to the N-terminal sequences of native Rgp and Kgp proteases were used to generate high-affinity MIP nanogels, taking advantage of our magnetic nanoparticle-based dispersed-phase imprinting approach. Analysis and understanding of these materials were undertaken using surface plasmon resonance (SPR). Once the performance was known, the sensor was exposed to the target proteins from complex media matrix of brain heart infusion (BHI), showcasing the effectiveness of a MIP nanogel-based SPR sensor towards biological matrices. A schematical representation is shown in Fig. 1.

## Methodology

### Materials

Iron(III) chloride hexahydrate (≥ 99%), sodium acetate (NaOAc, anhydrous), ethylene glycol (≥ 99%), aminopropyl trimethoxy silane (APTMS, 97%),* N*-tert-butylacrylamide (TBAM, 97%), *N*-isopropyl acrylamide (NIPAM, 97%), and ammonium persulfate (APS, ≥ 98%) were purchased from Merck. Sulfosuccinimidyl 4-(N-maleimidomethyl) cyclohexane-1-carboxylate (sulfo-SMCC) was purchased from Thermo Fisher. *N,N*-methylene bis(acrylamide) (BIS, ≥ 99%) was purchased from Alfa Aesar. N-(3-Aminopropyl) methacrylamide hydrochloride (APMA) was purchased from Polysciences Inc. N,N,N′,N′-tetramethylethylenediamine (TEMED) was purchased from FluoroChem. All chemicals were used without purification.

The designed Rgp and Kgp epitopes (> 95%) were purchased from Lifetein (USA). Bacterial culture supernatants E8, K1A, and W50-d were grown at and provided by the Section of Oral Biology and Pathology at Malmö University. The monomer FITC-acrylamide was prepared in-house.

### Preparation of peptide-immobilised magnetic nanoparticles

Magnetic nanoparticles (MNPs) were prepared using a one-pot solvothermal microwave method adapted from Sullivan et al. [27]. With FeCl_3_ˑ6H_2_O used as a single iron source, 5 g of FeCl_3_ˑ6H_2_O and 15 g of NaOAc were dissolved with magnetic stirring in 150 m of ethylene glycol to produce a stock solution. Five milliliters of this solution was added to a 10-mL microwave reaction vial (MRV). The MRV was sealed and then placed into a SmithCreator microwave oven (Personal Chemistry/Biotage) and the reaction was heated up to a temperature of 200 °C. The reaction was held at 200 °C for 20 min under pressure (12 bar). The resulting composite products were washed five times with deionised water followed by two washes with ethanol, and then collected with a magnet and finally dried for further use.

To a round bottom flask, 200 mg of dried MNPs was added with 100 mL of ethanol and milliQ water (3:1) and sonicated for 10 min to resuspend the MNPs. One milliliter of APTMS was added to form a thin silica shell and introduce primary amine groups on the surface. This mixture was sonicated for 2 h. Unreacted APTMS was removed by washing three times with ethanol. Three washes with milliQ water prepared the modified MNPs for the next modification step.

A solution of 4 mg of sulfo-SMCC was prepared in 400 µL of milliQ water and was added to 10 mL of amino-modified MNPs (20 mg/mL). The mixture was left to react for 1 h while shaking (400 rpm) at room temperature. Unreacted sulfo-SMCC was removed by washing three times with milliQ water. Next, 5 mL of these MNPs (20 mg/mL) was added to a vial and 1 mL of epitope solution (1 mg/mL) was added. The epitopes were left to immobilise onto the MNPs overnight while shaking (400 rpm) at room temperature. Finally, they were washed three times with milliQ water and concentrated to a final concentration of 25 mg/mL.

### Preparation of molecularly imprinted nanogels

An aqueous monomer solution containing TBAM (4 mM, 20% v/v ethanol), NIPAM (4.8 mM), BIS (0.2 mM), and APMA (1 mM) was added to a vial. Next, 250 µL of the FITC-monomer (1.6 mg/mL) was introduced to allow visual confirmation of the washing and elution steps later. Finally, 1 mL of the template-MNP solution was added to the monomer mixture and milliQ water was added to obtain a final volume of 10 mL. This whole mixture was left to shake at room temperature for 1 h to allow the functional monomers to arrange themselves around the template. Next, the mixture was purged with N_2_ gas for 30 min, after which 100 µL of APS (60 mg/mL) was added and the mixture was purged for an additional 5 min. To initiate the reaction, 100 µL of TEMED (6% v/v) was rapidly added, and the vial sealed and shaken overnight at 40 °C at a speed of 480 rpm.

Once the reaction was finished, the MNPs containing high-affinity nanogels were magnetically separated and low-affinity nanogels and unreacted materials were removed. To make sure everything was gone, the MNP and high-affinity nanogel mix were washed four times with milliQ water at 40 °C, until no fluorescence was observed in the supernatant. After the final wash, 4 mL of milliQ water was added to the vial, sonicated for several seconds, and placed at 4 °C for 4 h to allow for elution of high-affinity nanogels. In the middle, the mixture was again briefly sonicated before placing it back at 4 °C. After magnetic separation, the eluent was collected, and the vial refilled with 1 mL of milliQ water. This was placed at 4 °C overnight and the final eluent containing nanogels was collected.

### Characterisation of magnetic nanoparticles

Wide angle X-ray diffraction (WAXD) data were collected using a Xeuss 3.0 SAXS/WAXS laboratory-based instrument (Xenocs, Grenoble, France) at Malmö University (Malmö, Sweden). The X-ray beam was generated using a Cu Kα source (*λ* = 1.541 Å). The sample was exposed to vacuum during measurements in room temperature. The sample was sandwiched between two Kapton films (DuPontTM Kapton®, 0.013 mm thickness, Goodfellow, UK), one side being adhesive, sealing the particles between the Kapton films. The diffraction data were collected by the movable 3-axis WAXS detector (Pilatus3 R 300 K hybrid photon counting detector) at two different positions on a radial sample-to-detector distance (STDD) of 115 mm. These two positions covered the *q*-range 0.6 ≤ *q* (Å − 1) ≤ 4.54, where *q* is the scattering vector and is defined as:

$$\left|q\right|=q=\frac{4\pi }{\lambda }\text{sin}\theta$$

where 2*θ* is the scattering angle (2*θ*-range 7.5 ≤ 2*θ* (deg) ≤ 67.8). The *q*/*θ* scale was calibrated using lanthanum hexaboride. One-dimensional (1D) data was obtained by azimuthal averaging of the 2D diffraction patterns recorded by the detector. The data were corrected for background scattering (including the Kapton background), normalised to the direct beam, and merged using the Xenocs XSACT software (version 2.6). The exposure time of the sample/background was 300 s for each detector position.

A suspension of magnetic nanoparticles was pipetted as small droplets onto SEM aluminium sample stubs and allowed to dry. The stubs were sputtered with gold using an Agar automatic sputter coater at 30 mA, 0.08 mbar pressure and with a sputtering time of 40 s. SEM micrographs were obtained using a Zeiss EVO LS10 scanning electron microscope equipped with a LaB6 filament. Imaging was done in high vacuum mode using a secondary electron detector, at 15 kV accelerating voltage and 50 pA probe current. Imaging was done in high vacuum mode using a secondary electron detector, at 15 kV accelerating voltage, 50 pA probe current, and 5–6 mm working distance.

Fourier-transform infrared spectroscopy (FTIR) spectra were collected using a Nicolet 6400, equipped with a DTGS detector. The *smart*iTR accessory was used for the characterisation of dried modified magnetic nanoparticles. Five hundred spectra were collected at resolution 4. Compressed air was continuously run through the instrument during and before the measurements. Correction of the baseline and evaluation of data was performed using the OMNIC 6 software.

Dynamic light scattering measurements were performed to determine the hydrodynamic diameter of the synthesised nanogels. A Zetasizer Ultra (Malvern Panalytical) equipped with a He–Ne laser (688 nm) was set to backscatter mode, where measurements of samples were performed in triplicate at room temperature. Samples were first left overnight at 4 °C and then diluted 10 × with milliQ water prior to measurement. Data was analysed using the ZS Xplorer software.

### Characterisation of molecularly imprinted nanogels

Effective hydrodynamic diameters (*d*_*h*_) of the particles were determined by dynamic light scattering (DLS) with a Zetasizer Ultra (Malvern Panalytical) equipped with a He–Ne laser (688 nm) and set to backscatter mode, with measurements of samples performed in triplicate at 25 °C. Data was analysed using the ZS Xplorer software.

Concentrations of the nanogel solutions were calculated by taking 400 µL of the solution (in triplicate) and evaporating to dryness. The mass of the dried particles was then measured, and the amount multiplied by 2.5 to give the concentration in μg mL^−1^.

Affinity of the imprinted nanoparticles for the fluorescein target was investigated using a Reichert 2 SPR system (Reichert Technologies, Buffalo, USA) with an attached autosampler. Specificity of the imprinted nanomaterial was investigated by the binding of a non-target peptide of similar shape and size.

### Immobilisation of MIP nanogels onto the SPR sensor surface

A carboxymethyl dextran hydrogel-coated Au chip (Reichert, USA) was preconditioned within the SPR by use of a running buffer consisting of PBS (0.01 M) and 0.01% Tween 20 (PBST) at pH 7.4, at a flow rate of 10 µL min^−1^. The carboxylic acid groups on the dextran chip were activated with an injection of 1 mL of aqueous solution containing 40 mg EDC and 10 mg NHS passed over the chip (6 min at 10 µL min^−1^).

The MIP nanogels (approximately 300 µg) were activated by dissolving in 1 mL of 10 mM sodium acetate in PBST solution. This was injected over the left channel (working channel) of the chip for 1 min. The amine groups of the MIP nanogel react with the functionalised surface of the chip leading to covalent immobilisation of the nanoparticles. A quenching solution of ethanolamine (1 M at pH 8.5) was injected over both channels (working and reference) for 8 min, followed by a continuous flow of PBST at 10 µL min^−1^. All injections were taken from a stable baseline.

### Kinetic analysis using SPR

The kinetic analysis for the affinity of the target peptide (Rgp and Kgp) to the MIP nanogels was performed in a set pattern of a 2-min association (PBST with analyte in concentration range of 4–64 nM), 5-min dissociation (PBST only), and a regeneration cycle (regeneration buffer 10 mM glycine–HCl, pH 2 for 1 min) followed by a final stabilisation cycle (PBST for 1 min). An initial injection of blank PBST was used as the first run with increasing analyte concentration for subsequent runs. After the analyses were completed, signals from reference channel were subtracted from signals of the working channels. Selectivity of the MIP nanogels was investigated by repeating the kinetic analysis, but with a non-target analyte with the same concentration range (4–64 nM). All experiments were performed in replicate (*n* = 3).

The SPR responses were fitted to a 1:1 Langmuir fit bio-interaction (BI) model using the Reichert TraceDrawer software. The association rate constants (*k*_a_), dissociation rate constants (*k*_d_), and maximum binding (*B*_max_) were fitted globally, whereas the BI signal was fitted locally. Equilibrium dissociation constants (KD) were calculated by *k*_d_/*k*_a_.

To calculate the theoretical lower limit of detection (LOD) of the imprinted nanogels, a non-IUPAC method for determination was used. For each MIP nanogel/analyte epitope combination, a calibration curve was generated across the concentration range 4–64 nM, using the SPR fitted curve maxima. By using the line of best fit, the lowest detectable concentration (LOD) can be extrapolated and calculated at the intercept with the *x*-axis. These values are hypothetical and are therefore termed as the theoretical limit of detection (LOD).

## Results and discussion

### Rapid microwave-assisted synthesis of magnetic nanoparticles

Using a microwave-assisted approach, adapted from Sullivan et al., magnetic nanoparticles were produced in 20 min due to the accelerated heating of materials by dielectric heating effects. Microwave-assisted synthesis improves heating and energy efficiency because the energy produced by the microwave is only transferred directly to the reaction components, which are susceptible to microwave polarisation, minimising the time needed for the reaction to reach the activation energy, thus reducing the time needed for heating and minimising the occurrence of any unwanted side reactions and by-products.

Solvent choice is an important consideration for microwave-assisted synthesis as the solvent needs to be able to absorb microwaves and convert them into thermal energy. With this regard, ethylene glycol was the solvent of choice, due to its known high dissipation factor (tan *δ* = 1) and high boiling point. While the production of magnetic nanoparticles by a conventional solvothermal method can take over 8 h for the completion of the reaction, using this microwave-assisted methodology reduced this synthesis time down to 20 min.

**Fig. 2:**
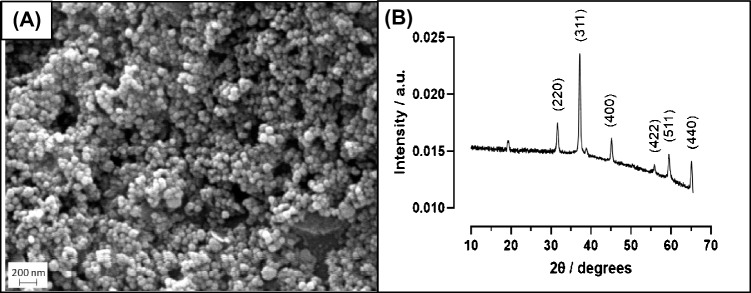
(**A**) SEM image of dried bare magnetic nanoparticles. 15 kV accelerating voltage, 50,000 × zoom. Image was sharpened and size measured using ImageJ. (**B**) XRD pattern of bare magnetic nanoparticles collected with an X-ray beam generated using a Cu Kα source (*λ* = 1.541 Å)

The SEM image for the magnetic nanoparticles is shown in Fig. 2A and demonstrates that the synthesised nanoparticles are spherical and dispersive with a diameter ranging from 40 to 50 nm.

The XRD pattern for the synthesised magnetic nanoparticles is shown in Fig. 2B and shows six distinctive strong diffraction peaks for the sample observed in the 2*θ* range of 20–80°, which are indexed as (220), (311), (400), (422), (511), and (440). Using the Scherrer equation (Eq. 1), the crystallite size of the magnetic nanoparticles can be estimated, with the equation relating the crystallite size (*D*_p_) and a specific diffraction peak broadening:

1 $${D}_{\text{p}}= \frac{K\lambda }{{\beta }_{311}\text{cos}{\theta }_{311}}$$

where *D*_p_ is the average crystallite site, *K* is the Scherrer constant (0.94), *β*_311_ is line broadening (full width at half maximum, in radians), and *θ*_311_ is the Bragg angle (radian). The estimated crystallite size for the Fe_3_O_4_ magnetic nanoparticles is shown to be approximately 41.03 nm and is consistent with the size estimates using SEM (40–50 nm). These results are consistent with iron oxide found in the inorganic crystal structure database (ICSD Collection Code 5247) [28] and confirm that Fe_3_O_4_ (magnetite) has been produced [29].

The synthesised magnetic nanoparticles showed their typical Fe–O vibration at 537 cm^−1^ in the corresponding FTIR spectrum (Figure S1). Other bands were visible in this spectrum due to incomplete removal of ethylene glycol. Amine functionalisation of the magnetic nanoparticles was achieved through the silanisation of the magnetic nanoparticles with APTMS. Characteristic bands for this modification are the Si–O stretching vibrations at 1051 cm^−1^ and the symmetric and asymmetric CH_2_ stretching vibrations at 2835 and 2912 cm^−1^. At 1375 cm^−1^, the symmetric methyl stretch of unreacted methoxy side groups can be seen.

### Template design and immobilisation

Templates for producing discriminative sites were chosen by comparing different sequence motifs of the two proteases. As in the case of antibody-antigen interactions, the epitope is typically a short solvent-exposed peptide sequence (8–20 amino acids) acting in this case as an antigenic determinant, thus constituting the site on the protein surface interacting with the MIP. Since the first demonstration of this approach and its combination with surface imprinting techniques, effective protocols for rational epitope imprinting are now available.

The most straightforward approach to find potential epitope candidates is to map out established immunogenic regions of the protein that can be accessed by the nanoMIP. Our first focus was to identify unstructured terminal sequences since these require no or minimal conformational stabilisation to complement the native protein structure.

Kgp and Rgp are 57 and 43 kDa cysteine proteases with pIs of 5.4 and 4.9 respectively (see SI). The work by Genco et al. has shown that immunisation of mice with the 20-amino acid N-terminal sequence of Rgp induced a protective immune response against *P. gingivalis* infection. These sequence motifs were therefore chosen as templates [30].

Following the amine functionalisation of the magnetic nanoparticles, we introduced a heterobifunctional tether (SMCC) [31]. It was anticipated that this would reduce detrimental surface-template interactions. Site-directed immobilisation of the peptide was then achieved by thiol-ene click coupling via the surface maleimide group and a terminal cysteine residue on the template peptide. The spectra of both peptides after coupling look similar in nature. In both cases, the amide I (C = O stretching vibration) and amide II (N–H bending and C-N stretching vibrations) bands at 1635 and 1533 cm^−1^ can be identified. The backbone of the peptide chain also shows up, evidenced by the asymmetric and symmetric CH_2_ stretching vibrations at 2851 and 2922 cm^−1^. Notable here is the shift in wavenumber, which is due to the molecular order. The CH_2_ stretching vibrations of the peptide point to a structure with higher molecular ordering compared to that of the APTMS modification [32]. This is potentially due to more random polymerisation direction of the APTMS. A clear difference in absorbance intensity can be seen at 879 cm^−1^, corresponding to the C–C bending vibration of the alkyl side chains belonging to the amino acids: alanine, valine, leucine, isoleucine, and methionine. The Rgp epitope contains 7 of these amino acids, while the Kgp epitope contains 9, causing the related higher intensity.

### Synthesis of molecularly imprinted nanogels

Using a magnetic dispersed-phase synthesis approach and a common monomer feed containing N-isopropylacrylamide (NIPAm, 48%), methylene-bis-acrylamide (BIS, 2%), N-tert-butylacrylamide (TBAm, 40%), and N-3-aminopropylmethacrylamide (APM, 10%), molecularly imprinted nanogels were produced for the target peptides. Using this synthetic approach produced MIP nanogel solutions of 625 µg mL^−1^ and 560 µg mL^−1^ for the Rgp and Kgp target peptides respectively. These concentrations reflect the enhanced nanoparticle yields obtained using this approach (> 100-fold per weight carrier) and match those of prior work.

The size of the nanogels was estimated using dynamic light scattering (DLS) and is presented in Figure S2. The *Z*-average diameters of the particles are shown to be 183 ± 1 nm and 137 ± 20 nm, with polydispersity index values (PDI) of 0.251 and 0.350, at 25 °C, for the Rgp epitope and Kgp epitope MIP respectively. The DLS curves shown in Figure S2 display a bimodal distribution, which is due to potential aggregation of the nanogels. Poly(*N*-isopropylacrylamide) is known to exhibit aggregating behaviour and is the main component of the nanogels [33]. This behaviour was taken into account, and to prevent aggregation issues during immobilisation onto the SPR chip, dried nanogels were redissolved in the PBST running buffer (PBS containing 0.01% Tween 20). Nonetheless, these DLS size results further support that this nanogel synthesis protocol produces regular homogenous particles.

Deposition of the nanogels onto the surface of the SPR chip (coated in a carboxymethyl dextran layer) is achieved through EDC/NHS coupling chemistry and is favoured because of the high percentage of amine-functionality within the nanogel. The pre-functionalisation of the gold SPR chips with a carboxymethyl dextran hydrogel layer allows for a good deposition profile of the nanogels, because of the ease of activation of the carboxyl groups on the hydrogel by the EDC/NHS. Ethanolamine is then used to deactivate any unwanted and unreacted carboxyl groups that are left on the SPR chip surface (after nanogel immobilisation), whilst also washing away any unbound nanogels. By using this selected deposition method, it is expected that only a single layer of the nanogels will be found on the chip surface, as the nanogels are unable to bind to themselves. During deposition, the nanogels are added in excess as this allows for maximum coverage on the chip, thus providing the best opportunity for the maximum population of available binding sites available per chip. With potentially having a theoretical maximal receptor (binding population) has allowed for the application of standardised models for ligand/receptor interactions, using a 1:1 kinetic model.

**Fig. 3:**
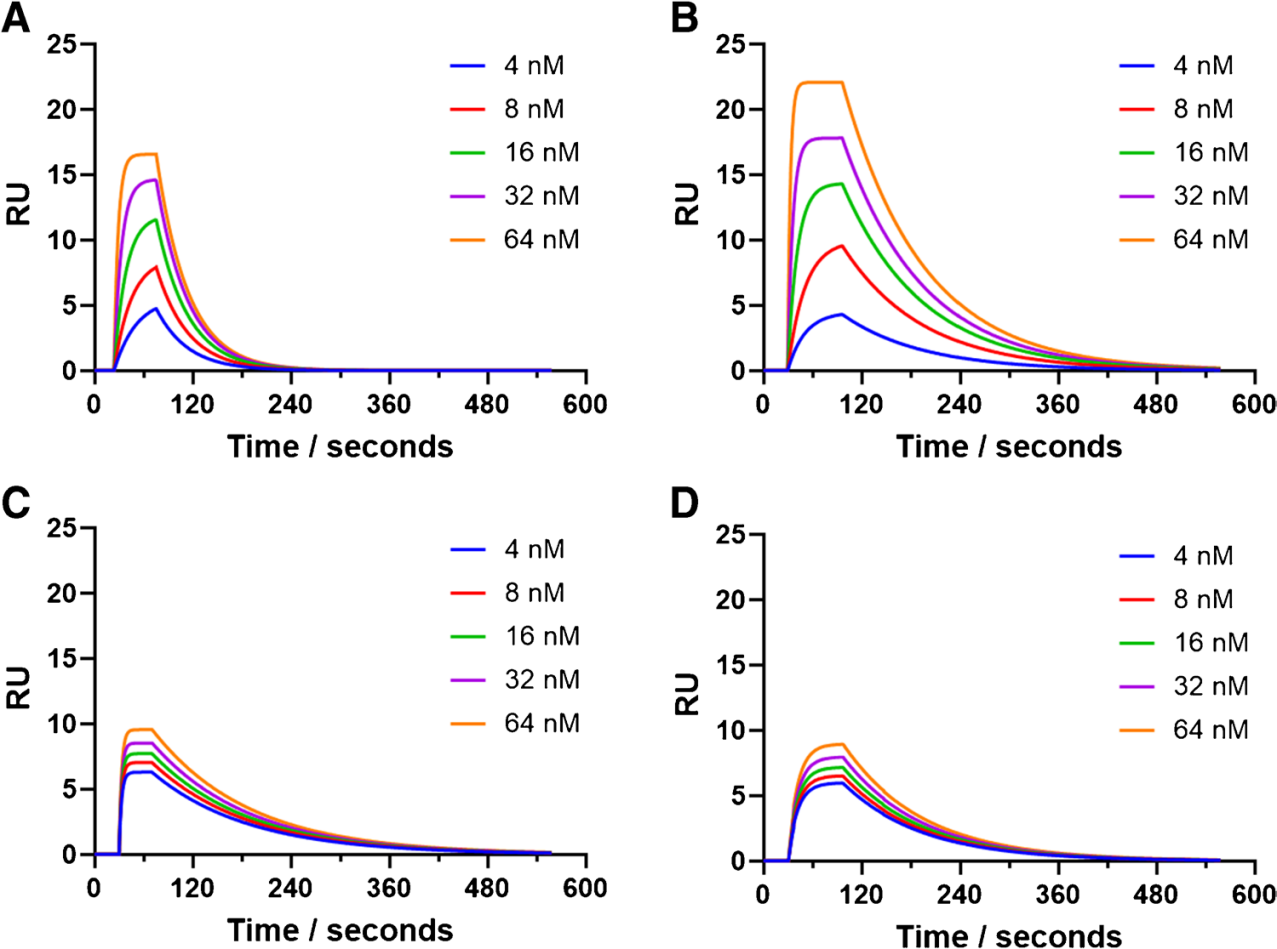
Representative fitted SPR curves showing the rebinding of the target and non-target peptides to the immobilised nanogel with five concentrations of the analyte in PBST. (A) Rgp binding to Rgp-imprinted nanogels; (B) Kgp binding to Kgp-imprinted nanogels; (C) Kgp binding to Rgp-imprinted nanogels; (D) Rgp binding to Kgp-imprinted nanogels

The SPR sensorgrams presented in Fig. 3 show the interactions of five different concentrations of the target peptides with their corresponding nanogels that were immobilised onto the surface of the sensor. The overall equilibrium dissociation constant (*K*_D_) values for the target interacting with their nanogels were extracted from the curves (Fig. 3) and the application of a 1:1 kinetic model, with these values summarised in Table 1.

**Table 1:** Calculated equilibrium dissociation constant (*K*_D_) of the nanogels from data presented in Fig. 3. All experiments were performed under ambient conditions and with three replicates

	*K*_D_ (nM)	
	Rgp	Kgp
Rgp nanogel	79.4 (± 5.6)	6430.0 (± 8.4)
Kgp nanogel	5310.0 (± 280.0)	89.7 (± 6.4)

The interactions of the Rgp and Kgp peptides and their corresponding nanogels (Fig. 3A and B, respectively) were calculated with the *K*_D_ values of 79.4 nM and 89.7 nM for the Rgp and Kgp peptides, respectively (Table 1). This is consistent with previously published values for this type of nanogels that have been imprinted for peptides [34]. Bognár et al. produced nanogels for the target peptides of SARS-CoV-2 RBD (peptide sequence GFNCYFP), which gave *K*_D_ values of 60 nM and shows that the nanogels produced in this study are also comparable to those of monoclonal antibodies.

The specificity of the nanogels was explored by investigating the cross-reactivity and non-specific binding of the nanogels to the binding of non-target peptides. This is presented in Fig. 3C and D, for the non-target peptide with Kgp peptide binding to the Rgp nanogel and Rgp peptide binding to the Kgp nanogel, respectively. The *K*_D_ values calculated with the non-target to the nanogels were shown to be in the micromolar range (Table 1) which shows an approximate 80-fold and 60-fold decrease in affinity, for the Rgp and Kgp nanogels, respectively. It clearly shows target specificity, consistent with cross-reactivity observations with similar produced nanogels [35, 36].

**Fig. 4:**
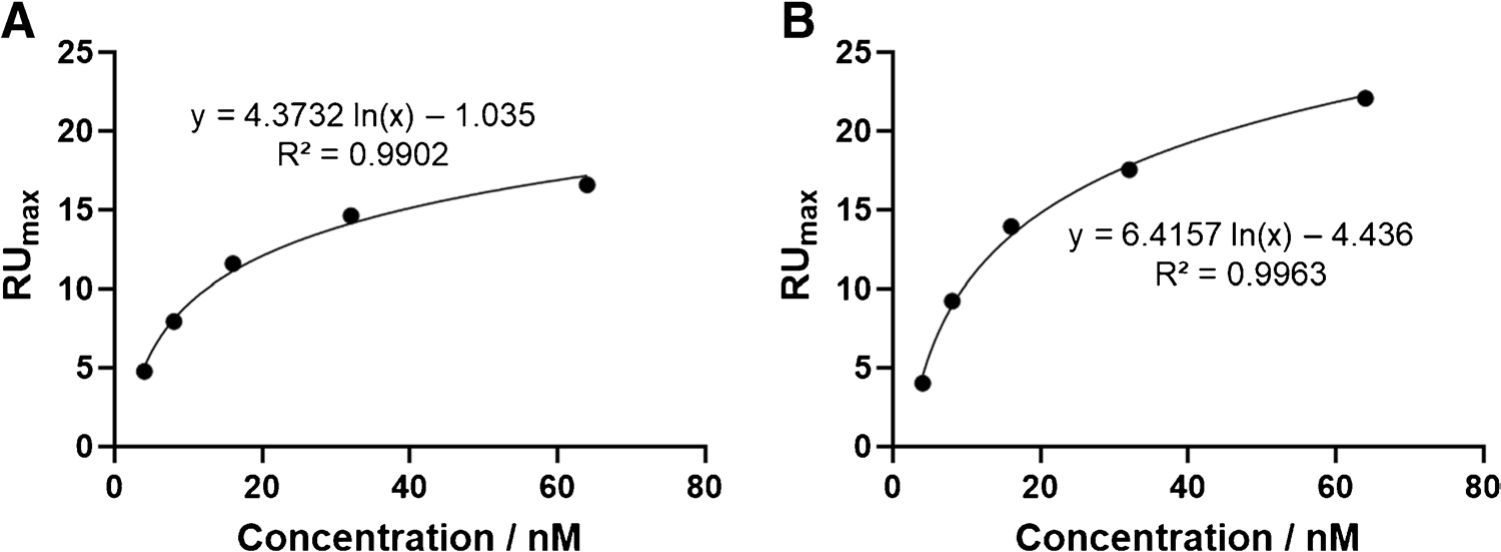
Representative calibration curves for the elution of limit of detection of SPR-based sensor showing the relative signal vs concentration. (**A**) Rgp binding to Rgp-imprinted nanogels; (**B**) Kgp binding to Kgp-imprinted nanogels

Through retrospective elution of signal vs concentration from the sensorgrams presented in Fig. 3, concentration calibrations were plotted (Fig. 4) and used to calculate theoretical lower limits of detection (LOD) (Table 2), which can then be used for SPR-based biosensor generation. It should be noted that these calibration curves appear to produce negative RU values when the concentration is zero (i.e., a blank). However, that is an artefact following subtraction of the control channel from the working channel when establishing an accurate and tangible baseline.

**Table 2:** Calculated theoretical lower limits of detection (LOD) of the nanogel SPR-based sensor from data presented in Fig. 4

	Lower limit of detection (LOD) (nM)	
	Rgp	Kgp
Nanogel	1.3	2.0

Using the calibration plots presented in Fig. 4, theoretical lower LODs can be calculated, by using the line of best fit equation, whereby when *y* equals 0, *x* equals the theoretical LOD. However, this method of calculating the LOD deviates from the typically used standard method, such as 3 *S*/*N*. We opted for the method described here since (i) using the standard method results in LOD values higher than the actual physical lowest measured concentration; (ii) the blank signal from the reference channel is subtracted from those signals obtained in the working channels, leading to no available blank value to be used in the standard method; and (iii) the method used here has been described before in literature [36]. It should be noted that the LOD values obtained through this method are hypothetical values and we therefore refer to them as the *theoretical LOD*. The calculated theoretical lower LODs (shown in Table 2) for the SPR-based sensors are 1.3 nM for the Rgp nanogel sensor and 2.0 nM for the nanogel sensor. These values are consistent with each other and consistent with other nanogel SPR-based sensors for proteins [22] showing that the robust sensors produced in this work are highly sensitive for the detection of *P. gingivalis* proteases and offer the potential for a new, cost-effective, and robust biosensor alternative. Furthermore, these LODs are lower than the presence of Rgp and Kgp proteases found in gingival crevicular fluids of patients diagnosed with periodontal disease, which is predicted to be between 7 nM and 1.5 µM [37].

**Fig. 5:**
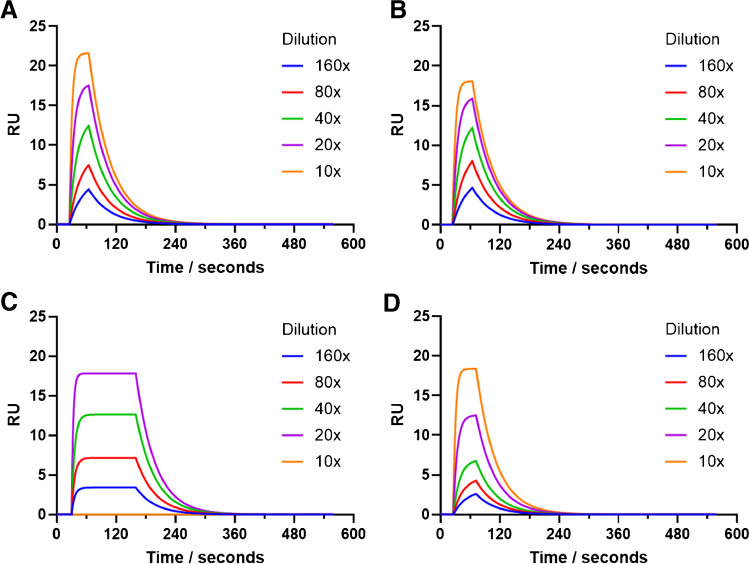
Representative sensorgrams of interactions of the imprinted nanogels immobilised onto a carboxymethyl dextran hydrogel-coated gold SPR chip for five concentrations of the K1A, E8, and W50-d proteins, from a BHI media. (**A**) K1A bacterial culture supernatant binding to Rgp-imprinted nanogels; (**B**) E8 bacterial culture supernatant binding to Kgp-imprinted nanogels; (**C**) W50-d bacterial culture supernatant binding to Rgp-imprinted nanogels; (**D**) W50-d bacterial culture supernatant binding to Kgp-imprinted nanogels

At present, there is a lack of accurate quantification techniques to provide reliable detection of Rgp and Kgp, hence the need for new biosensors and detection techniques. Therefore, within the building of a biosensor for *P. gingivalis*, detection using bacterial culture supernatants E8 (Rgp knockout), K1A (Kgp knockout), and the native W50-d (wild-type) in brain heart infusion (BHI) media matrix was investigated. These samples were diluted in the PBST running buffer that is also used for calibration through a reference channel. The surface on this reference channel did not contain any immobilised Rgp or Kgp nanogels, thus allowing signal subtraction from those channels containing the nanogels. In doing so, any potential signal effects caused by the biological matrix are removed and we can show the true value of interactions between the target molecules and the nanogels. Figure 5 shows the SPR sensorgrams for the interactions of these protein targets, with the corresponding nanogels, from BHI media matrix. These samples are of unknown concentrations; thus, the kinetics and sensorgrams (Fig. 5) are presented from a series of dilutions. From these sensorgrams, an estimate for the protein (E8, K1A, and W50-d) concentrations is calculated using the calibrations (Fig. 5) and presented in Table 3. Important to note here is that BHI is a nutrient-rich growth medium that is commonly used for culturing bacteria. The presence of peptones and salts provide an environment that could interfere with rebinding between target and recognition sites of the MIP in a similar fashion as a serum-rich matrix or gingival crevicular fluid would. Peptones are a mix of protein hydrolysates and therefore consist mainly of amino acids and short peptide sequences [38]. Since short sequence epitopes were used in the imprinting procedure, it becomes clear that competition arises between the peptones of the BHI medium and the bulkier protein targets. Ionic interactions between template and functional monomers were used in the synthesis of the MIPs. The presence of salts in the BHI medium can influence the ionic interactions between the target and recognition sites.

**Table 3:** Estimate protein (E8, K1A, and W50-d) concentrations calculated from the Rgp and Kgp epitope nanogel sensors

Estimated concentration (nM)			
K1A protein (Rgp sensor)	E8 protein (Kgp sensor)	W50-d protein (Rgp sensor)	W50-d protein (Kgp sensor)
3.4 (± 0.3)	4.4 (± 0.9)	3.5 (± 0.3)	3.7 (± 1.8)

It should be acknowledged that non-His-tagged reference proteins are not commercially available; hence, we were unable to perform the relevant spike-in experiments and hence explains the use of the bacterial culture supernatants. The wild-type W50-d bacterial culture is one of the strains that belong to the *P. gingivalis* species, and therefore produces both Rgp and Kgp proteases. E8 and K1A are knockout strains of the same species used to investigate more specifically the binding of Kgp and Rgp, respectively. While performing this analysis using real-life samples would be optimal, this is approximated by using the W50-d wild-type strain, which is one of the species found within gingival crevicular fluid in patients [39]. However, this study clearly demonstrates that detection of *P. gingivalis* proteases from complex media is achievable using the nanogels as a synthetic recognition material.

The estimated concentration values (Table 3) calculated from the sensorgrams (Fig. 5) and calibrations curves (Fig. 4) show the K1A protein (using the Rgp nanogel-based sensor) to be 3.4 nM and the E8 protein (using the Kgp nanogel-based sensor) to be 4.4 nM. These values are low and are consistent with other nanogel SPR-based sensors for proteins [22]. This study shows that, for the first time, the production of MIP nanogels using small epitopes for periodontal disease biomarkers is capable of achieving high selectivity with low LODs that are similar to MIPs produced for other proteins. This comparison between using epitopes and proteins is necessary due to epitope imprinting still being relatively new in this field. Furthermore, the detection of the W50-d (wild-type protein), which contains both Rgp and Kgp proteases, was 3.5 and 3.7 nM for both the Rgp and Kgp nanogel-based sensors, respectively. The comparable concentration estimates using two different SPR nanogel-based sensors (Rgp and Kgp nanogel-based sensors) show consistency and accuracy with the sensors produced using this methodology. Typically, *P. gingivalis* is only detected in 25% of healthy patients, while it is detected in 80% of a group of patients diagnosed with periodontitis [40]. Current detection methods exist with LODs of approximately 0.02 to 1.1 nM, though these detect the enzymatic activity and require longer incubation times [41–43]. The work presented here is comparable to what is currently available, albeit with a more robust recognition material. This highlights that new robust sensors produced in this work are highly sensitive for the detection of *Porphyromonas gingivalis* proteases and offer the potential for a new, cost-effective, and robust biosensor alternative. The fact that detection of these proteases was in BHI media matrix further highlights the versatility of these biosensors in detecting a specific target from complex media without biological fouling, which is a potential problem of antibody-based biosensors [44].

## Conclusion

Here, we can demonstrate the selective recognition of *P. gingivalis* proteases from complex media using an epitope imprinting process producing MIP nanogels. Using an adaptive solid-phase protocol, magnetic nanoparticles were first produced using a unique, green, and efficient microwave synthesis method. These particles offer a consistently small solid-phase that, after template immobilisation, offers a greater surface area and larger template to solid-phase ratio than is usually seen with the glass-bead approach. The nanogels produced using this method displayed high affinity (*K*_D_ values of the target analyte in nM range) towards the target, while also displaying a high degree of selectivity (*K*_D_ values of non-target analyte in the µM), which is consistent with previous studies for similar targets, as well as producing a nanogel material with affinity that is comparable with monoclonal antibodies.

Using these materials, we were able to create an effective and robust sensor for the detection of low concentrations of the target analytes from a complex media of BHI media matrix. This was achieved by immobilising the nanogels onto a SPR gold chip and producing a SPR sensor platform, utilising optical sensor affinity testing. Calculated theoretical lower LOD values using these sensors show detection of the target proteases in the lower nanomolar range and are significantly relevant for the detection of *P. gingivalis* proteases from biological samples. This highlights the strength of the imprinting process and further demonstrates the effectiveness of utilising the nanogel materials as a viable alternative to mono/polyclonal antibodies as cost-effective and robust recognition materials.

While the system highlights the power of SPR as a sensing platform, it currently only offers a single target detection. As MIP nanogels offer a great degree of flexibility for their use in a variety of platforms, we are now exploring the integration of these materials into a range of new platforms, including electrochemical platforms as well as multiplex systems and immunoassays. This would truly showcase the power and effectiveness of these synthetic recognition materials in a variety of practical applications.

## Supplementary Information

Below is the link to the electronic supplementary material.

Supplementary file1 (DOCX 135 KB)
