# Supplementary material for: Molecularly imprinted nanogels as synthetic recognition materials for the ultrasensitive detection of periodontal disease biomarkers
Source: Anal Bioanal Chem. 2024 Jun 20;416(30):7305–16. doi: 10.1007/s00216-024-05395-6 (PMC11584468; doi:10.1007/s00216-024-05395-6)
Supplement: Supplementary file 1 — Supplementary file1 (DOCX 135 KB) [file 216_2024_5395_MOESM1_ESM.docx]

**Molecularly imprinted Nanogels as Synthetic Recognition Materials for the Ultrasensitive Detection of Periodontal Disease Biomarkers**

Thomas Hix-Janssens^1^, Julia R. Davies^2^, Nicholas W. Turner^3^, Börje Sellergren^1*^, Mark V. Sullivan^3*^

^1^ Department of Biomedical Science, Faculty of Health and Society, Malmö University, 205 06 Malmö, Sweden

^2^ Section for Oral Biology and Pathology, Faculty of Odontology, Malmö University, 205 06 Malmö, Sweden

^3^  Department of Chemistry, Dainton Building, University of Sheffield, Brook Hill, Sheffield S3 7HF, United Kingdom.

*mark.sullivan@sheffield.ac.uk

* [borje.sellergren@mau.se](mailto:borje.sellergren@mau.se)

**Supplementary Data**

The sequence of the RgpB recombinant protease is shown below. It consists of 244 amino acids, of which the epitope used is coloured in red. It is 20 amino acids long and capped with a cysteine. The theoretical pI of this epitope is 9.5, with a molecular weight of 2.4 kDa.

**YTPVEEKENG RMIVIVPKKY** EEDIEDFVDW KNQRGLRTEV KVAEDIASPV TANAIQQFVK QEYEKEGNDL TYVLLVGDHK DIPAKITPGI KSDQVYGQIV GNDHYNEVFI GRFSCESKED LKTQIDRTIH YERNITTEDK WLGQALCIAS AEGGPSADNG ESDIQHENII ANLLTQYGYT KIIKCYDPGV TPKNIIDAFN GGISLANYTG HGSETAWGTS HFGTTHVKQL TNSNQLPFIF DVAC

The sequence of the Kgp protease is shown below. It consists of 366 amino acids, of which the epitope used is coloured in red. It is 20 amino acids long and capped with a cysteine. The theoretical pI of this epitope is 4.2, with a molecular weight of 2.3 kDa.

**DVYTDHGDLY NTPVRMLVVA** GAKFKEALKP WLTWKAQKGF YLDVHYTDEA EVGTTNASIK AFIHKKYNDG LAASAAPVFL ALVGDTDVIS GEKGKKTKKV TDLYYSAVDG DYFPEMYTFR MSASSPEELT NIIDKVLMYE KATMPDKSYL EKALLIAGAD SYWNPKIGQQ TIKYAVQYYY NQDHGYTDVY SYPKAPYTGC YSHLNTGVGF ANYTAHGSET SWADPSLTAT QVKALTNKDK YFLAIGNCCV TAQFDYPQPC FGEVMTRVKE KGAYAYIGSS PNSYWGEDYY WSVGANAVFG VQPTFEGTSM GSYDATFLED SYNTVNSIMW AGNLAATHAG NIGNITHIGA HYYWEAYHVL GDGSVM


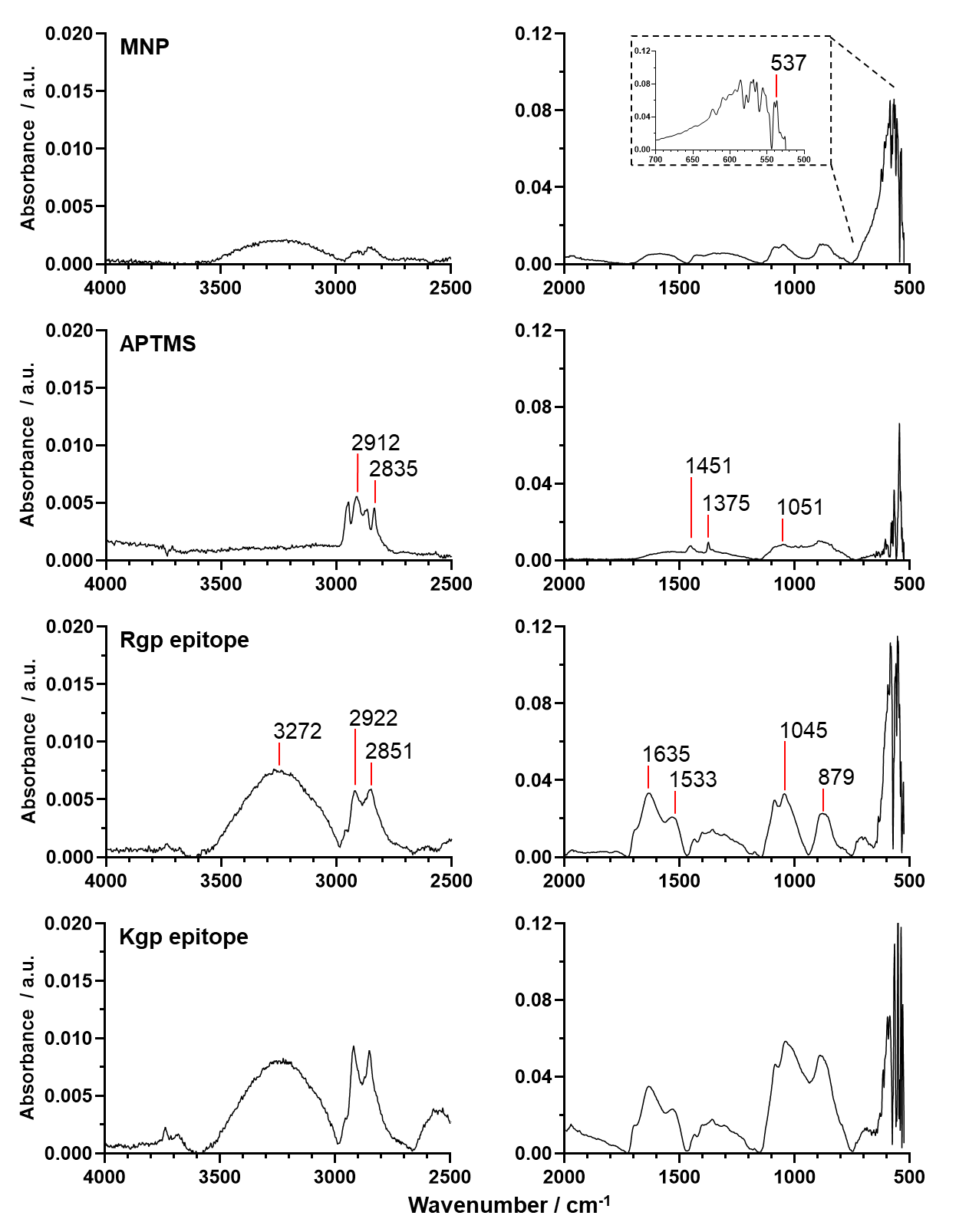


**Figure S1**. FTIR spectra of bare magnetic nanoparticles (MNP), modified with aminopropyl trimethoxysilane (APTMS), and after immobilisation of the Rgp and Kgp epitopes.

**
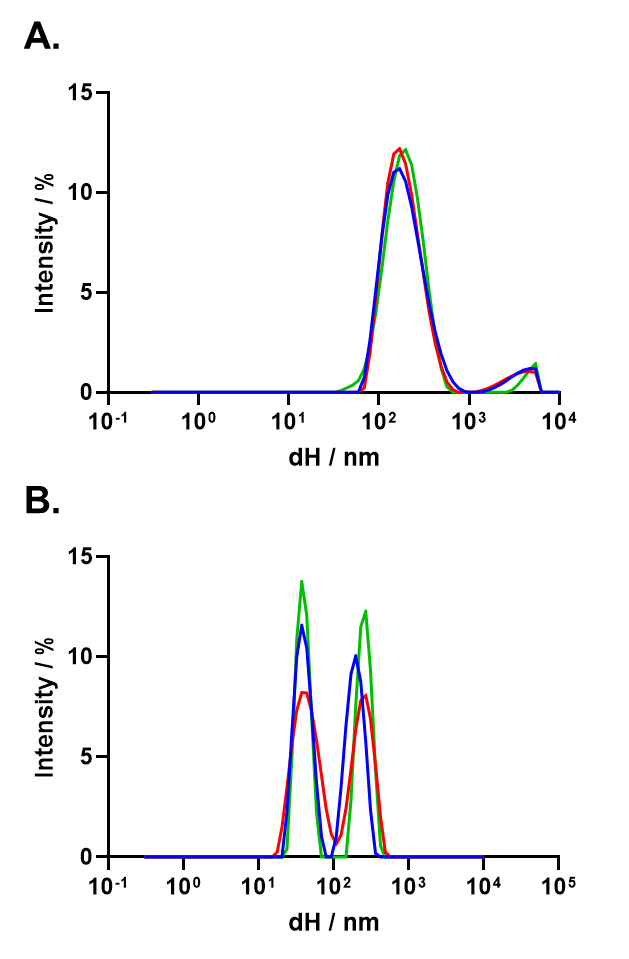
**

**Figure S2.** DLS size distribution by intensity of (A) the Rgp MIP and (B) the Kgp MIP.
